# Chimeric CTLA4-CD28-CD3z T Cells Potentiate Antitumor Activity Against CD80/CD86–Positive B Cell Malignancies

**DOI:** 10.3389/fimmu.2021.642528

**Published:** 2021-04-02

**Authors:** Shouheng Lin, Lin Cheng, Wei Ye, Shanglin Li, Diwei Zheng, Le Qin, Qiting Wu, Youguo Long, Simiao Lin, Suna Wang, Guohua Huang, Peng Li, Yao Yao, Xiaofang Sun

**Affiliations:** ^1^ Department of Obstetrics and Gynecology, Key Laboratory for Major Obstetric Diseases of Guangdong Province, The Third Affiliated Hospital of Guangzhou Medical University, Guangzhou, China; ^2^ Key Laboratory of Reproduction and Genetics of Guangdong Higher Education Institutes, Guangzhou, China; ^3^ State Key Laboratory of Respiratory Disease, Guangdong Provincial Key Laboratory of Stem Cell and Regenerative Medicine, Guangzhou Institutes of Biomedicine and Health, Chinese Academy of Sciences, Guangzhou, China; ^4^ Bioland Laboratory (Guangzhou Regenerative Medicine and Health Guangdong Laboratory), Guangzhou, China; ^5^ Department of Respiratory Medicine, Nanfang Hospital, Southern Medical University, Guangzhou, China

**Keywords:** immunotherapy, CAR-T, CTLA4, CD80, CD86, myeloid-derived suppressor cells

## Abstract

The adoptive transfer of chimeric antigen receptor T (CAR T) cells have been recognized as a promising therapeutic strategy for the treatment of hematological malignancies; however, clinical success using CAR T cells for the treatment of solid tumors are still limited since the T-cell function is inhibited by negative signals in the microenvironment of solid tumors. CTLA4 is a well-known immune checkpoint molecule, thus we developed a novel CAR by converting this negative signal to positive signal. The CAR developed consists of the extracellular and transmembrane domains of CTLA4 and the cytoplasmic domains of CD28 and CD3z (CTLA4-CAR T). CTLA4-CAR T cells exhibited superior cytokine secreting activities and cytotoxic to tumor cells *in vitro* and in xenograft models. CTLA4-CAR T cells were found to accumulate in tumors and are toxic to myeloid-derived suppressor cells (MDSCs) without signs of severe GVHD and CRS in preclinical models. Thus, this chimeric CTLA4-CAR can enhance the antitumor activity of CAR T cells and shed light on the strategy of using armed CAR T cells to target the immunomodulatory tumor microenvironment.

## Introduction

T cell responses can be compromised by the presence of negative costimulatory signaling molecules, such as programmed death-1 (PD1), T cell Ig mucin-3 (TIM3), Lymphocyte activation gene-3 (LAG3), and cytotoxic T lymphocyte-associated antigen 4 (CTLA4) (1–3). The CTLA4-CD86/CD86 axis is a well-known immune checkpoint inhibitor pathway. CTLA4, which is up-regulated after T cell activation (2), is homologous to the T cell costimulatory protein CD28, and bind to its ligands CD80 and CD86 on many tumors and on cells within the tumor microenvironment (TME), such as antigen-presenting cells, B cells, macrophages, and the stromal cell subset (4). The CTLA4-CD80/CD86 pathway serves as a negative feedback mechanism to control the immune responses to inflammatory stimuli (5). CTLA4-CD80/CD86 signal initiates T cells anergy or exhaustion, which reduces the activities of T cells, whereas blockade of the interaction between CTLA4 and its ligands reverses effector T cells exhaustion, thereby reinforcing anti-tumor activities of T cells (6). Recent preclinical and clinical evidence has shown promise in treating cancer by utilizing anti-CTLA4 antibodies (7–10); however, the clinical trial for solid tumors achieved very limited success because of the weak anti-tumor T cell immune responses, it is very challenging to establish the effective anti-tumor response in solid tumors due to its immunosuppressive TME.

In recent years, chimeric antigen receptor T (CAR T) cell therapies have shown exciting therapeutic modalities for some difficult cancer (11–13). CARs generally comprise an extracellular ligand recognition domain, typically a single-chain variable fragment (ScFv) fused to the signaling domain of CD3z (14). Second-generation CARs contain another intracellular costimulatory domain, which may include CD28, 4-1BB, ICOS, CD40, and CD27 molecules, to enhance cytokine secretion and proliferation of CAR T cells (15–17). Many CARs have been developed to recognize multiple tumor-associated antigens (TAAs); however, currently available targets for CAR T cell therapy are still limited due to high heterogeneity among cancer patients (18–20). In addition, tumor relapse occurred in many patients who achieved disease remission after initial CAR T cell therapy, which may have been due to the loss of tumor antigen on tumor cells (21).

Targeting of CTLA4-CD86/CD86 interaction by the administration of blocking antibodies could enhance the potency of immunotherapy for cancers. However, partial or poor complete response (CR) was observed in patients, suggesting that the therapeutic effects of a naked antibody would not be potent enough for the curable treatment of cancer (22, 23). Previous studies had reported that CTLA4-CD28 chimera gene-modified T cell, which the intracellular signaling domain of CTLA4 was replaced with the CD28 signaling domain, showed significantly enhanced anti-tumor effect in murine tumor models (24, 25). Therefore, in this study, we attempted to convert the negative CTLA4-CD86/CD86 signal by generating a novel CTLA4 signaling pathway in T cells to disrupt the interaction of immune checkpoint inhibitors CTLA4 and CD80/CD86 to effectively treat CD80/CD86-expressing cancer. To this end, we developed a novel chimeric receptor that can recognize CD80 and CD86 as tumor antigens on malignant B cells by combining the extracellular and transmembrane domains of CTLA4 with the intracellular signaling domains of CD28 and CD3z and explored its potential in cancer treatment.

## Materials and Methods

### Human Tissues

Cord blood samples and primary B lymphoma samples were obtained according to procedures approved by the institutional review boards at Nanfang Hospital (Guangdong, China). The use of human tissue samples in this study was approved by the Committee for the Ethical Review of Research Involving Human Subjects at Nanfang Hospital, Southern Medical University. Institutional guidelines regarding human experimentation were followed, according to the Helsinki Declaration of 1975. The protocol was approved by the Ethical Committee of The Third Affiliated Hospital of Guangzhou Medical University. Written informed consent was obtained from individual or guardian participants.

### Mice

Animal experiments were performed in the Laboratory Animal Center of the Guangzhou Institutes of Biomedicine and Health (GIBH), and all animal procedures were approved by the Animal Welfare Committee of GIBH. NOD-*SCID-IL2Rg* ^−/−^ (NSI) mice were derived at the Laboratory Animal Center of GIBH. C57BL/6J mice were purchased from Vital River Laboratory Animal Technology Co. (Beijing, China). Mice were maintained in specific pathogen-free cages and provided with autoclaved food and water. Adult male mice aged 6 to 8 weeks were used in this study. Protocols were approved by the relevant Institutional Animal Care and Use Committee (IACUC).

### Cell Cultures

HEK-293T, Platinum-E, and B16F10 cells were maintained in Dulbecco’s modified Eagle’s medium (Gibco, Grand Island, NY, USA) supplemented with 10% fetal bovine serum (FBS; Gibco, New York, NY, USA). All lymphoma cell lines (NALM6, RL, Raji, and K562 cells) were purchased from American Type Culture Collection (ATCC; Maryland, USA) and were labeled with green fluorescent protein (GFP) and luciferase. Cells were cultured in RPMI-1640 medium (Gibco, New York, NY, USA) supplemented with 10% fetal bovine serum (FBS; Gibco, New York, NY, USA). All cells were cultured at 37°C in an atmosphere of 5% carbon dioxide.

### Flow Cytometry

Peripheral blood, spleen, liver, and bone marrow from mice were treated with red blood cell lysis buffer (Biolegend) before staining. Solid tissue samples were mechanically chopped with scalpels, placed in culture medium (DMEM with 5% FBS, 0.5 mg/ml collagenase A, 0.2 mg/ml hyaluronidase V, and 0.02 mg/ml DNase I), and digested for 45 min at 37°C. The resulting suspensions were resuspended in PBS, and the cells were pelleted at 300 r.c.f. for 3 min.

All antibodies were purchased from BD Biosciences unless otherwise noted. Flow cytometric analysis was performed using an Accuri C6 or LSRFortessa cell analyzer (BD Biosciences, San Jose, CA), and data were analyzed using FlowJo software (FlowJo, LLC, Ashland, OR, USA). Anti-human CD80 PE-conjugated (560925), anti-human CD86 APC-conjugated (560956), anti-mouse CD11b FTTC-conjugated (561688), anti-mouse Ly6C PerCP-Cy5.5-conjugated (560525), and anti-mouse Ly6G PE-Cy7–conjugated (560601) antibodies were used for analyses. 4′,6-Diamidino-2-phenylindole (DAPI; 0.1 µg/ml final concentration; Invitrogen, D1306) was used to distinguish live and dead cells. Staining was performed on ice for 30 min, and cells were then washed with PBS containing 2% FBS before cytometry analysis.

### Vector Design

**Figure 1:**
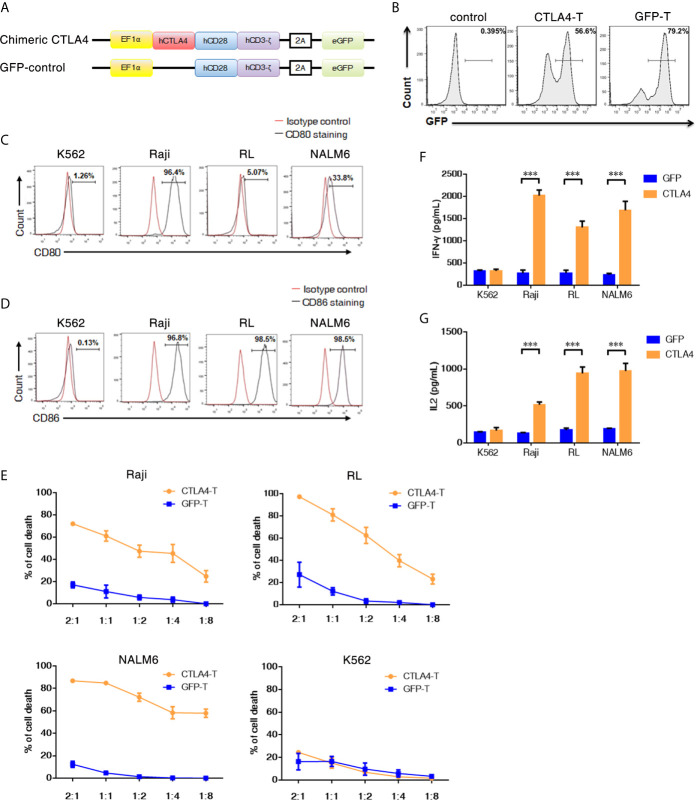
T cells transduced with the CTLA4-CD28-CD3z chimeric gene showed enhanced *in Vitro* Cytotoxicity. **(A)** The chimeric CTLA4 molecule contains the extracellular and transmembrane domains of human CTLA4, the cytoplasmic signaling region of human CD28, and the intracellular domain of human CD3z. GFP was used to fluorescently label the cells. **(B)** Representative flow cytometric analysis of the transduction efficiency of chimeric CTLA4 or GFP (control) in human activated T cells that were transduced with a lentivirus. CTLA4-T: CTLA4 chimera-transduced T cells, GFP-T: GFP-transduced T cells. **(C, D)** Representative flow cytometric analyses of CD80/CD86 expression in K562, Raji, RL, and NALM6 cells. **(E)** Activated T cells transduced with either chimeric CTLA4 or GFP (control) and cocultured with the indicated cell lines for 18 h, mean ± SD. The levels of IFN-γ **(F)** and IL-2 **(G)** secreted into the culture supernatant were measured by ELISA with a 1:1 E:T ratio, mean ± SD, unpaired two-tailed t-test. Significance values: ***P < 0.001.

The human CTLA4 CAR comprises the extracellular and transmembrane portions of human CTLA4, the cytoplasmic region of human CD28, and the intracellular domains of human CD3z (**Figure 1A**). All the domains were synthesized by GenScript (Nanjing) Co., Ltd. (Nanjing, China). The mouse chimeric CTLA4, comprising the extracellular and transmembrane domains of mouse CTLA4, the cytoplasmic region of mouse CD28 and the intracellular domain of mouse CD3z (**Figure 3A**), was synthesized by GenScript (Nanjing) Co., Ltd. (Nanjing, China). The human CTLA4-chimeric gene was cloned into lentiviral pWPXLD expression vectors, the murine CTLA4-chimeric genes were cloned into pMX expression vectors. All the sequences used in experiments were confirmed by automated DNA sequencing.

### Production of Lentivirus and Retrovirus

The lentivirus plasmids and another two packaging plasmid (psPAX2 and pMD2.G) were transduced to HEK-293T cells by using polyethyleneimine (Sigma-Aldrich, St. Louis, MO, USA). After 48 and 72 h, the supernatant containing lentivirus was harvested and filtered by using 0.45-μm syringe filter. The retrovirus plasmid was transduced to Platinum-E cells by using polyethyleneimine (Sigma-Aldrich, St. Louis, MO, USA). After 48, the supernatant containing retrovirus was harvested and filtered by using 0.45-μm syringe filter.

### Isolation, Transduction, and Expansion of Primary Human T Lymphocytes

Peripheral blood mononuclear cells (PBMCs) were isolated from cord blood using Lymphoprep (StemCell Technologies, Canada) according to the manufacturer’s instructions. Primary human T cells were isolated from PBMCs *via* negative selection by using a pan-T Isolation Kit (Miltenyi Biotec, Germany). Isolated T cells were maintained in RPMI-1640 medium supplemented with 10% FBS (Biochrom, Australia), 10 mM HEPES, 100 IU/ml recombinant human IL-2, 2 mM glutamine, and 1% penicillin-streptomycin (Gibco, New York, USA). T cells were stimulated with an ImmunoCult™ Human CD3/CD28 T Cell Activator (StemCell Technologies, Canada) for 48 h. T cells were transfected with CAR vector lentiviral supernatants in the presence of 8 μg/ml polybrene at a multiplicity of infection of 2.0 (Sigma-Aldrich, St Louis, USA). Twelve hours after transfection, T cells were cultured in a fresh medium containing IL-2 (300 U/ml); subsequently, a fresh medium was added every 3 days to maintain cell density within the range of 0.5 to 1 × 10^6^ cells/ml. CAR-T cells determined by flow cytometry at day 5, as GFP+, and then were included in the experiments.

### Isolation, Transduction, and Expansion of Primary Mouse T Lymphocytes

Mouse spleens were removed from euthanized C57BL/6J mice, and mouse T cells were enriched by using a mouse pan-T Isolation Kit (Miltenyi Biotec, Germany). Isolated T cells were incubated in medium supplemented with human IL-2 (10 ng/ml, PeproTech, Rocky Hill, USA) and human IL-7 (2 ng/ml, PeproTech, Rocky Hill, USA) and were cocultured with mouse T cell activator beads (Miltenyi Biotec, Germany) for 48 h and then transduced with retrovirus by centrifuging at 1,200*g* for 90 min and then placed in a cell culture incubator (37°C, 5% CO_2_) for 24 h. CAR-T cells determined by flow cytometry at day 5, as GFP+, and then were included in the experiments.

### Cytotoxicity Assays

Target cells (NALM6-GL, RL-GL, and Raji-GL) were cocultured in triplicate wells U-bottomed 96-well plates with T cells that expressed either human CTLA4 CAR or GFP-CAR (control) at the indicated effector cell:target cell (E:T) ratios. Target cell viability was monitored 18 h later by adding 100 µl/well of the substrate D-luciferin (potassium salt) (Cayman Chemical, Michigan, USA) at 150 µg/ml. Background luminescence was negligible (<1% of the signal from wells containing viable target cells alone). Percent viable target cells (%) was calculated as (experimental signal − blank signal)/(targeted signal − blank signal) × 100, and percent cytotoxicity as 100 − percent viable target cells.

### Cytokine Enzyme-Linked Immunosorbent Assay (ELISA)

After 18 h of co-culture cells at a 1:1 E:T ratio, the supernatants were collected, and the levels of interferon γ (IFN-γ) and interleukin-2 (IL-2) in the supernatant were detected using cytokine ELISA kits (e-Bioscience, San Diego, CA, USA) according to the manufacturer’s instructions.

### Xenograft Models of Malignant B Cells

NOD/SCID/IL2Rg^−/−^ mice were subcutaneously injected with NALM6, RL, or Raji cells (2 × 10^5^) with 10% Matrigel (20 µl Matrigel in 180 µl PBS) on day 0. Two days after transplantation, the mice were randomly divided into three groups (five mice/group) and injected *via* the caudal vein with CTLA4-T cells, GFP-T cells, or non-transduced T cells (2×10^5^ cells) in 200 µl PBS. Tumor volume was calculated every 7 days. The tumor length and width were measured every 5 days using a Vernier caliper, tumor volume was calculated using the following equation: (length × width^2^)/2.

For primary lymphoma models, primary lymphoma tumors were resected and placed in RPMI-1640 medium in an ice bath. The tumors were diced into 3 × 3 × 3 mm cubes and subcutaneously transplanted into NOD/SCID/IL2Rg^−/−^ mice. When the tumor volume in the PDX model mice reached 50 to 100 mm^3^, the animals were intravenously injected with CTLA4-CAR T cells, GFP-CAR T cells, or non-transduced (2×10^5^ cells). After 30 days, all the mice were sacrificed, and the tumors were assessed.

For autologous transplantation, 6- to 8-week-old CD45.2 B6 mice were used as recipients. Mice were radiated by 4.5 Gy and subcutaneously injected with B16F10 cells (2 × 10^5^) with 10% Matrigel (20 µl Matrigel in 180 µl PBS) on day 0. A week after transplantation, the mice were randomly divided into three groups (five mice/group) and injected *via* the caudal vein with CTLA4-CAR T cells, GFP-CAR T cells (2 × 10^5^ cells), or PBS, donor T cells were derived from CD45.1 B6 mice. Tumor volume was calculated every 7 days. After 35 days, all the mice were sacrificed, and the tumors were assessed. For long-term observation, the mice were raised until tumor volume exceeded 2,000 mm^3^.

The severity of systemic graft-versus-host disease (GVHD) developed in the mice was assessed according to a mouse clinical GVHD scoring system as a previous report (26). Weight loss of <10% was scored 0, 10% to 25% was scored as 1, > 25% was scored as 2. For gastrointestinal symptoms, the scoring system denoted 0 as normal and 1 as suffering from diarrhea. For posture and activity, the scoring system denoted 0 as normal, 1 for hunching at rest and a mild to moderate decrease in activity, and 2 for severe hunching and a severe decrease in activity. For fur texture and skin integrity, the scoring system denoted 0 as normal, 1 for mild to moderate fur ruffling and scaling of the paws and tails, and 2 for severe fur ruffling and an obviously denuded mouse. Each clinical GVHD score was measured twice a week.

For the CRS Model, 6- to 8-week-old female CB17.B6-Prkdc^scid^ Lyst^bg^/Crl (SCID-beige) mice (Charles River) were intraperitoneally injected with 3 million Raji cells and tumors left to grow for 21 days. Mice were injected intraperitoneally with 30 million CAR T cells in PBS, weight change was measured every 8 h, the levels of the inflammatory cytokines were analyzed at 24 and 48 h, mice were euthanized and analyzed 3 days after T cell transplantation.

### Immunohistochemical Staining and Analysis

Paraffin-embedded sections were deparaffinized and, after heat-mediated antigen retrieval, stained with an antibody against GFP protein (ab290, 1:500) overnight at 4°C. The sections were incubated with a peroxidase-labeled antibody at 37°C for 60 min. The slides were then stained with DAB and counterstained with hematoxylin. All slides were imaged with a microscope (DMI6000B; Leica Microsystems), results were analyzed using Image-Pro Plus 6.0 software.

### Statistical Analysis

Samples and animals were random allocation and were unbiasedly included in the analysis unless specific mention. All cell culture experiments, real-time PCR, and ELISA were performed in triplicate at least three independent times. Statistical analysis to determine group differences was done by Student’s t-test (two groups) or ANOVA analysis with Tukey’s multiple comparison test (three or more groups) using GraphPad Prism, version 7.0 (GraphPad Software), all statistical analyses are described in the figure legends. All cell culture experiments were performed in triplicate at least three independent times. The p values are considered as follows: *p<0.05, **p<0.01, and ***p<0.001.

## Results

### T Cells Transduced with the CTLA4-CD28-CD3z Chimeric Gene Showed Enhanced *In Vitro* Cytotoxicity

To alter the negative signals of CTLA4 in T cells, we attempted to convert the negative signals to the positive ones by linking the intracellular stimulatory domains from CD28 and CD3z with CTLA4 to generate a CTLA4-CD28-CD3z CAR (CTLA4-CAR). For the CTLA4-CAR plasmid, the extracellular and transmembrane domains from human CTLA4 were designed to be fused to the intracellular signaling domains from human CD28 and CD3z (**Figure 1A** and **Supplementary Figure 1A**). The corresponding cDNA was cloned into a lentiviral vector and transduced efficiently into activated human T cells, chimeric CTLA4 expression was analyzed in the GFP+ cell population. The transduction efficiencies of CTLA4-CAR were found to be approximately 50% to 60% (**Figure 1B** and **Supplementary Figure 1B**).

Studies have shown that the CD80 (B7.1) and CD86 (B7.2) ligands of CTLA4 are highly expressed in malignant B cells (27, 28). The expression of either CD80 or CD86 is likely to facilitate co-stimulation *via* CTLA4 chimeras. Therefore, we detected the surface expression levels of CD80 and CD86 in Pre-B acute lymphoblastic leukemia (ALL) (NALM6) and B cell lymphoma (Raji and RL) cell lines. Flow cytometry revealed that both CD80 and CD86 were highly expressed in Raji cells, CD86 was highly expressed in NALM6 and RL cells, which exhibited low levels of CD80 expression. Neither CD80 nor CD86 was expressed in an erythroid precursor cell line K562 cells (**Figures 1C, D**).

To determine whether CTLA4-chimera-transduced T cells (CTLA4-T) could specifically recognize and kill CD80-positive or CD86-positive tumor cells, we performed cytotoxicity assays by co-culturing CTLA4-T cells or genetically modified control T cells (GFP-T) with the tumor cells. CTLA4-T cells efficiently lysed CD80-positive or CD86-positive tumor cells (Raji, RL, and NALM6), but not CD80/CD86-negative K562 cells, whereas control effector cells (GFP-T) could not initiate specific lysis on either cell line (**Figure 1E** and **Supplementary Figure 1C**). In line with these observations, CTLA4-T cells secreted higher levels of IFN-γ and IL-2 than GFP-T cells when coculturing with Raji, RL, and NALM6 cells, no significant difference in cytokine secretion was observed in co-cultures of CAR-T cells and K562 cells (**Figures 1F, G** **)**. These results suggested that modified CTLA4-T cells retained significant cytotoxic activities toward CD80/CD86-positive tumor cells specifically.

### CAR T Cells Redirected to CD80/86 Significantly Suppress the Tumorigenesis of Subcutaneous Xenografts

**Figure 2:**
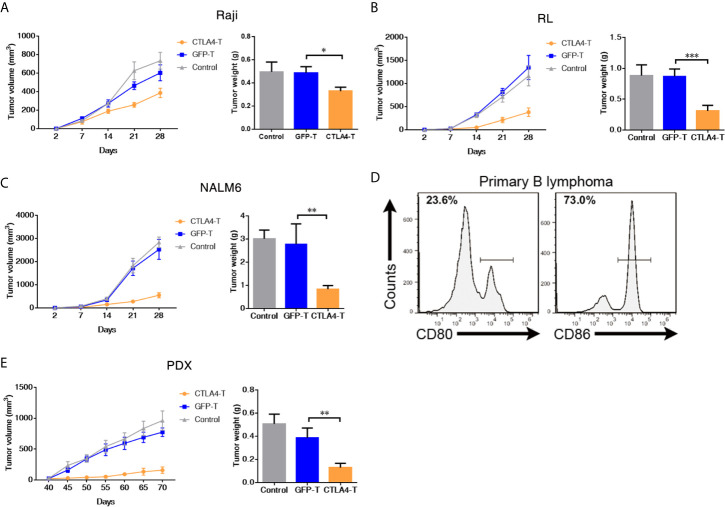
CAR T cells redirected to CD80/86 significantly suppress the tumorigenesis of subcutaneous xenografts. **(A–C)** NOD/SCID/IL2Rg^−/−^ mice were subcutaneously injected with 2 × 10^5^ of Raji, RL, or NALM6 cells and were intravenously administered human T cells transduced with either chimeric CTLA4 or GFP. Blank control groups comprised mice intravenously administered non-transduced T cells (2 × 10^5^ cells, five mice/group). The tumor weight of the Raji, RL, and NALM6 xenografts was weighed after 28 days. The tumor volumes in the CDX models were measured and calculated every 7 days. **(D)** Representative flow cytometric analyses of CD80 and CD86 expression in a xenograft comprising tumor cells from a B cell lymphoma patient. **(E)** NOD/SCID/IL2Rg^−/−^ mice were subcutaneously transplanted with patient-derived xenografts (PDXs) of B cell lymphoma to create PDX mouse models, which were treated with CTLA4-T, GFP-T, or non-transduced T cells when the tumor volume reached 50 to 100 mm^3^. The total number of GFP-positive T cells injected per mouse was 2 × 10^5^. Tumors in the mice in all three groups (five mice/group) were weighed at the end of the experiment. The tumor volume was measured and calculated every 5 days. Data are shown as the mean ± SD from independent experiments. One-way ANOVA; significance values: *P < 0.05; **P < 0.01; ***P < 0.001.

To examine the *in vivo* anti-tumor activity of CTLA4-T cells toward CD80/CD86-expressing tumors, we developed subcutaneous xenograft models using NOD/SCID/*IL2Rg* ^−/−^ mice. Raji, RL, or NALM6 cells were subcutaneously implanted into the mice 2 days before initiating immunotherapy. Experimental mice received CTLA4-T cells, GFP-T cells, or non-transduced T cells (control group), and observation for 4 weeks. The potent anti-tumor effect was observed in mice treated with CTLA4-T cells, whereas GFP-T cells did not suppress tumor growth, at the end of the experiment, all mice treated with CTLA4-T cells had significantly decreased tumor volume and tumor weight (**Figures 2A–C**), whereas mice in GFP-T cells treated or the control groups developed large tumors.

The PDX model is an effective tool for preclinical research (29–32). To further assess the anti-tumor efficacy of CTLA4-T cells against primary tumors, we developed B cell lymphoma PDX mouse models by using primary tumor tissues with high CD80 and CD86 expression (**Figure 2D**). In line with the cell line experiments, the mice treated with CTLA4-T cells displayed more pronounced tumor regression than those treated with GFP-T cells or treated with PBS (**Figure 2E**). Taken together, these results suggested that CTLA4-T cells confer strong anti-tumor activities and could specifically suppress CD80/CD86-expressing tumors *in vivo*.

### Murine CTLA4-Chimeric T Cells Are Toxic to MDSCs Within Tumors

**Figure 3:**
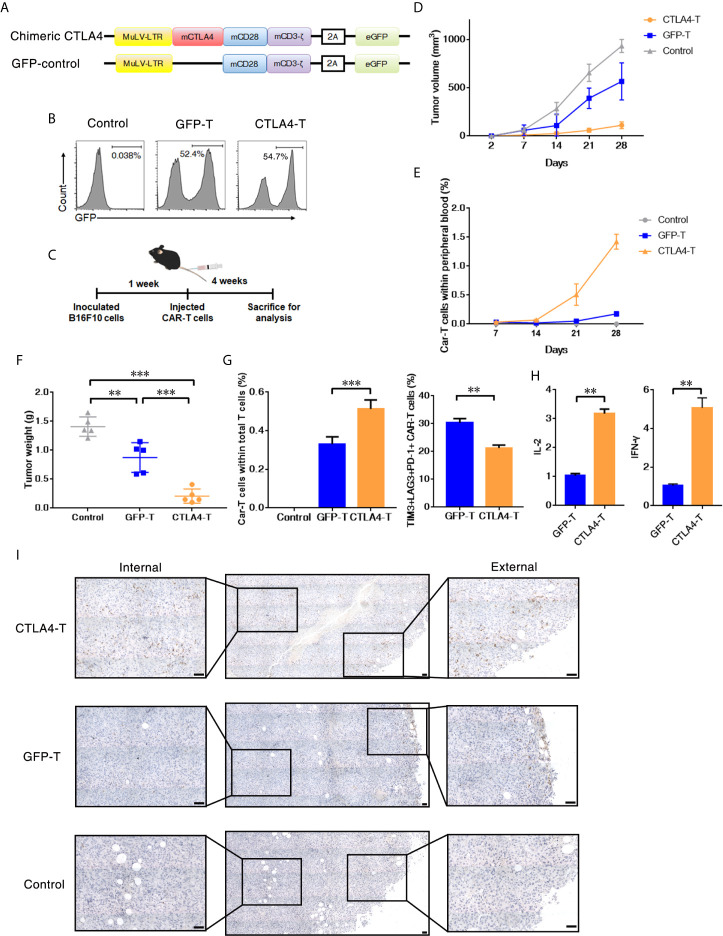
T cells expressing the CTLA4-CD28-CD3z chimera had effective tumor infiltration. **(A)** Murine chimeric CTLA4 molecules contained the extracellular and transmembrane domains of mouse CTLA4, the cytoplasmic region of mouse CD28, and the intracellular domains of mouse CD3z. T cells expressing GFP were constructed as the control group. **(B)** Representative flow cytometric analysis of murine chimeric CTLA4 or GFP expression in mouse T cells. **(C)** Experimental scheme for evaluating murine CTLA4-CAR T cells efficacy, 2 × 10^5^ of B16F10 cells were subcutaneously transplanted, and mice were intravenously administered T cells transduced with either chimeric CTLA4 or GFP or PBS (Control), five mice/group. **(D)** The tumor volumes in the mice were measured and calculated every 7 days. **(E)** The percentages of CAR T cells in peripheral blood of the mice were measured and calculated every 7 days. **(F)** The B16F10 tumor weight was weighed after 35 days, mean ± SD, one-way ANOVA. **(G)** The percentages of CAR T cells in total infiltrated T cells within the tumor tissues, and the percentages of TIM3+LAG+PD-1+ CAR T cells, mean ± SD, one-way ANOVA. **(H)** qRT-PCR analysis of the mRNA expression of the indicated genes. The results were normalized to glyceraldehyde 3-phosphate dehydrogenase (GAPDH) mRNA levels and are presented as the mean ± SEM (n = 3), unpaired two-tailed t-test. **(I)** Immunohistochemical staining identified the infiltrated CAR T cells in resected tumors, GFP+ cells were stained. Significance values: **P < 0.01; ***P < 0.001.

We further investigated whether CTLA4-T cells targeting mouse CD80/CD86+ cells were safe and effective in an autologous transfer setting. We constructed a murine CTLA4-chimeric molecule, which comprised the extracellular and transmembrane domains of murine CTLA4, murine CD28 costimulatory domain, and murine CD3z signaling domain (**Figure 3A** **and** **Supplementary Figure 2A**). The transduction efficiencies of murine CTLA4-CAR were found to be approximately 55% (**Figure 3B**). Murine T cells expressing either the CTLA4 chimera or GFP were transplanted into C57BL/6J mice bearing B16F10 tumors. 4 weeks after transplantation, the mice were euthanized for analysis (**Figure 3C**). In autologous recipients, CTLA4-T cells significantly suppressed tumor growth (**Figure 3D**). Moreover, in peripheral blood the percentages of remaining CAR T cells in the CTLA4-T group were higher, compared to the GFP-T group (**Figure 3E**), suggesting that the converted CTLA4 signal improved the persistence of CAR T cells *in vivo*. At the experimental endpoint, mice treated with CTLA4-T cells had significantly decreased tumor weight (**Figure 3F**). The infiltration of T cells was validated in the tumor tissues, CTLA4-T cells were accumulated in residual tumors (**Figure 3G**). Also, fewer LAG3+TIM3+PD-1+ T cells were detected in the tumor tissue of the CTLA4-T group (**Figure 3G**, **Supplementary Figure 2B**). The expression of IL-2 and IFN-γ were up-regulated in the CTLA4-T cells (**Figure 3H**), which indicated advanced fitness of the CTLA4-T cells. Moreover, we found intratumoral infiltration of CTLA-4 T cells, whereas no specific infiltrated T cells were detected on staining in the sections of tumors treated with GFP-T cells (**Figure 3I**). CD4/CD8 ratio shifted after T cell transplantation, CD8+ CTLA4-T cells were growing faster *in vivo*, higher CD8+ T cell percentage was associated with higher anti-tumor efficacy (**Supplementary Figure 2C–D**).

**Figure 4:**
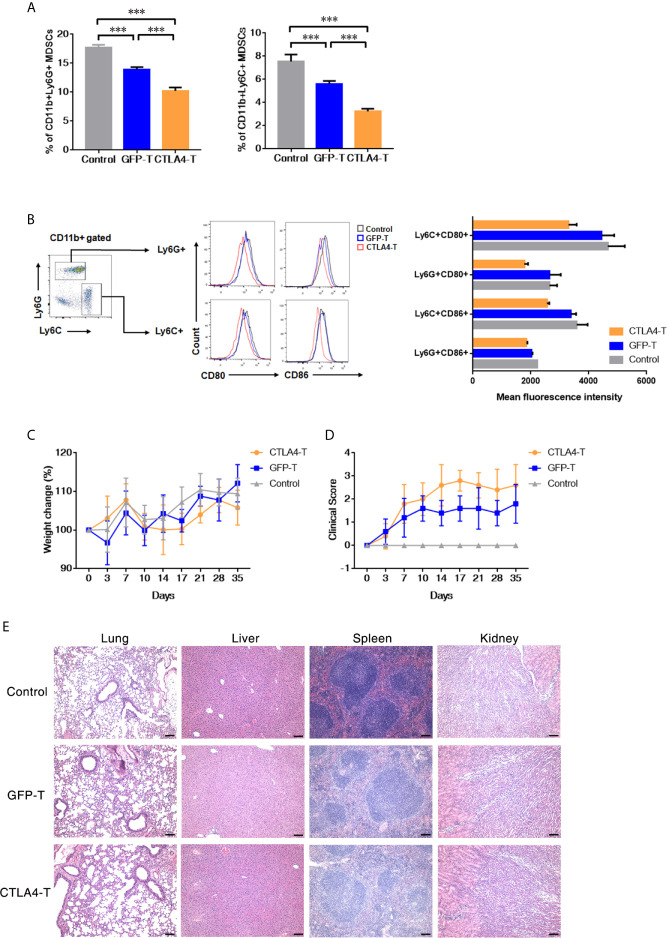
Murine CTLA4-chimeric T cells show toxicity against MDSCs. **(A)** Statistical analysis of MDSCs percentage with tumors by flow cytometry, mean ± SD, one-way ANOVA. **(B)** Representative flow cytometric analysis of the expression of CD80 and CD86 on MDSCs after CTLA4-CAR T cell therapy. **(C)** Weight change of autologous mice (n = 5) after T cell transfer. **(D)** Mice were monitored for GVHD pathology score twice a week. **(E)** H&E staining of organs, scale bar, 100 μm. Significance values: ***P < 0.001.

Myeloid-derived suppressor cell (MDSC) is a population of immature myeloid cells with immune suppressive function, these cells are also expressing CD80 and CD86 (33, 34). We further explored the anti-MDSCs potential of CTLA4-T cells, both granulocytic MDSCs (G-MDSCs, CD11b+Ly6G+) and monocytic MDSCs (M-MDSCs, CD11b+Ly6C+) were decreased within tumor tissues following CTLA4-T cells administration (**Figure 4A**). CD80/CD86-positive MDSCs were targeted by CTLA4-T cells (**Figure 4B**). These observations indicate that targeting MDSCs is responsible for some, if not all, of the enhanced intratumoral infiltration and anti-tumor activity of CTLA4-T cells.

Although no significant weight loss was observed in autologous recipients (**Figure 4C**), we found the T cell inoculated mice showed signs of few Graft-versus-host disease (GVHD). Using the GVHD clinical scoring system, the clinical status of the T cell inoculated mice were scored. Assessing the clinical GVHD manifestations, the mice with CTLA4-T cells transfusion showed a mild hunching posture and a ruffling fur texture, suggesting the occurrence of GVHD (**Figure 4D**). The histopathology of lung, liver, spleen, and kidney tissue was assessed, no significant morphological changes were observed (**Figure 4E**).

**Figure 5:**
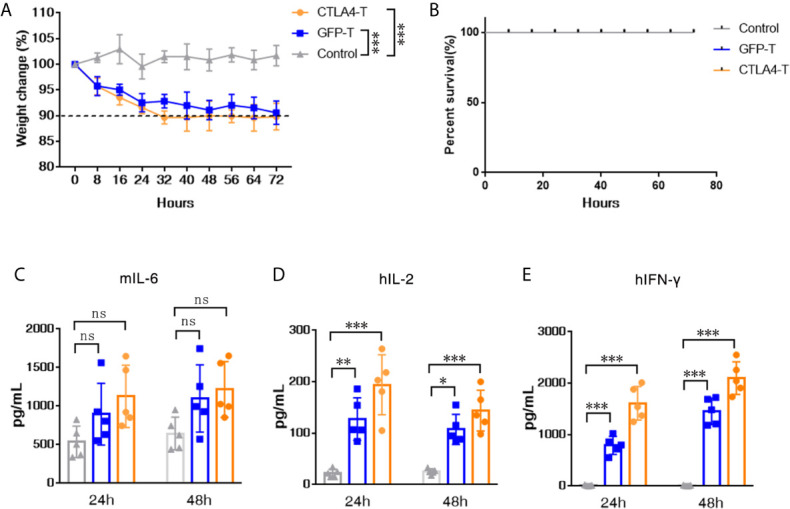
CTLA4-chimeric T cells elicited mild CRS *in vivo*. **(A)** Weight change of tumor-bearing mice (n = 5) after T cell transfer, two-way ANOVA with Tukey’s multiple comparison test. **(B)** Percent survival of mice after CAR T cell transfer (n=5 mice for each group). **(C–E)** Serum levels of mIL-6, hIL-2, and hIFN-γ 24 and 48 h post CAR T cell transfer were measured by ELISA. Significance values: *P < 0.05; **P < 0.01; ***P < 0.001; ns, non-significance.

To evaluate the potential for CTLA4-T cells to elicit the cytokine release syndrome (CRS) in mice, we established high tumor burden xenograft models as reported in prior studies (35–37). We found that human CTLA4-T cells elicited acute inflammatory responses associated with piloerection, malaise and, weight loss in recipients (**Figure 5A**). No death due to CRS happened in mice infused with CTLA4-T cells (**Figure 5B**), though high levels of mouse IL-6, an indicator of CRS (38), were detected with CTLA4-T cells and GFP-T cells (**Figure 5C**). The levels of the inflammatory cytokines hIL-2 and hIFN-γ in the serum were higher in the mice with CTLA4-T cells transfusion (**Figures 5D, E** **)**. Taken together, these results suggest that CTLA4-T cells may have the potential to elicit CRS or GVHD.

## Discussion

The expression of CD80/CD86 on tumor cells and immunosuppressive cells with the TME, such as MDSCs, makes the CTLA4-CD80/CD86 axis a remarkable target for cancer immunotherapies. In this study, we generated CD80/CD86-targeted CAR T cells to destroy CD80/CD86-associated tumor cells in culture and in the tumor xenograft mouse models. Our findings revealed that the cytotoxic activities of CTLA4-T cells against tumor cells were associated with their targeting specificity. CTLA4-T cells had enhanced abilities of TME infiltration, tumor suppression, and targeted MDSCs in tumor xenograft mouse models. These results rationalized the extension of our CD80/CD86-targeted CAR T cell-based immunotherapy for human malignant B cell lymphoma treatment.

CD80 and CD86 are highly expressed in approximately two-thirds of a cohort of 70 malignant B cell samples from patients with non-Hodgkin lymphoma. Thus, CTLA4-chimeric T cell therapy could be a promising approach to treat B cell malignancies. In clinical reports, B cell lymphoma patients who have received standard therapy may suffer from a relapse concomitant with increased expression of the costimulatory molecule CD86 (28). Currently, Patel et al. observed the expansion of CTLA4+PD-1− T cells close to CD86+ tumor cells and tumor-associated macrophages (39), which suggests that the interaction between CD86 and CTLA4 might be a key negative regulator in Hodgkin lymphoma. However, CTLA4 blockade using ipilimumab (anti-CTLA4) failed to show clinical benefits in high-risk cancer patients (40).

Immunotherapy using CAR T cells is in its infancy, its efficacy against leukemia has been widely recognized (41). However, clinical success using CAR T cells for the treatment of solid tumors was still limited. Clinical trials based on CAR targeting single antigens, such as human epidermal growth factor receptor 2 (HER2) (42), mesothelin (MSLN) (43), prostate stem cell antigen (PSCA) (44) are found mixed results due to the loss of tumor antigen on tumor cells, which confirms a fundamental issue with CAR T cell therapies: a lack of ideal single antigen targets. Another major barrier for CAR T cell therapies in solid tumors is the immunosuppressive TME (45). Thus, reports recommended that combining CAR T cell therapies with immune checkpoint inhibitors, such as CTLA4 and PD-1 inhibitors. Whereas our strategy of utilizing CTLA4-targeted CAR T cells has the advantages of not only suppressing cancer cells but also blocking immune checkpoint. For other CD80/CD86-negative solid tumors, it is worthy of consideration to utilize CAR construct targeting the tumor-associated antigens as well as converting CTLA4 signals. Besides, adoptive transfer of CTLA4-chimeric CAR T cells can avoid the loss of the negative regulation of the entire body’s immune system caused by anti-CTLA4 antibodies (25). This response may reduce the risk of developing an autoimmune disease for tumor patients (46, 47). Therefore, treating with CTLA4-CAR T cells as an alternative therapeutic regimen may be a promising option for these patients.

Our study showed the effects of CTLA4-CAR T cells on MDSCs cytotoxicity. Although it shed light on the strategy of using armed CAR T cells to target immunomodulatory TME, it also indicated that CTLA4-CAR T cells showed toxicity against non-malignant CD80/CD86 expressing cells. We observed mild GVHD in autologous recipients of CTLA4-T cells, high tumor burden xenograft models of CAR T cell-induced CRS suggested that CTLA4-T cells have the potential to elicit CRS. Therefore, CTLA4-T cells may present a risk for clinical development even in the autologous setting. Additional preclinical and clinical studies would be needed to validate the safety of CTLA4-T cells.

The current proof-of-concept study provides support of using armed CAR T cells for targeting immunomodulatory TME not only for CD80/CD86-CTLA4 axis but also for PD-1/PD-L1 (48), TIM3 (49), LAG3 (50). Our results indicate that converting the negative CTLA4-CD86/CD86 signal leads to improved antitumor activity without eliciting severe GVHD and CRS, suggesting that CTLA4-CAR could be employed to improve CAR T cell efficacy.

## Data Availability Statement

The original contributions presented in the study are included in the article/**Supplementary Material**. Further inquiries can be directed to the corresponding authors.

## Ethics Statement

The animal study was reviewed and approved by Guangzhou Institutes of Biomedicine and Health, Chinese Academy of Sciences.

## Author Contributions

SHL, LC, and WY contributed to the conception and design, the collection and/or assembly of data, data analysis and interpretation, and manuscript writing. SLL, DZ, GH, and LQ contributed to the provision of study material or patient samples and the collection and/or assembly of data. QW, YL, SiL, and SW provided animal care and administrative support. PL, YY, and XS contributed to the conception and design of the study, data analysis and interpretation, manuscript writing, and the final approval of the manuscript and provided financial support. All authors contributed to the article and approved the submitted version.

## Funding

This work was supported by Strategic Priority Research Program of the Chinese Academy of Sciences (XDB19030205), National Key Research and Development Plan (2017YFE0131600, 2019YFA0111500), National Natural Science Foundation of China (81961128003; 81972672; 31872800; 81773301; 82003054; 81870121); and China Postdoctoral Science Foundation (2018M640771), Guangdong Provincial Significant New Drugs Development (2019B020202003), Guangdong Basic and Applied Basic Research Foundation (2019A1515110084, 2019A1515010062, 2020A1515011516), Guangdong Special Support Program (2017TX04R102), Science and Technology Planning Project of Guangdong Province (2017B030314056), Natural Science Foundation of Guangdong Province (2020A0505100062), Guangdong Provincial Key Lab of Translational Medicine in Lung Cancer (2017B030314120), Guangzhou City Science and Technology Key Topics Project (201904020025), Guangzhou Science and Technology Plan Project (201907010042, 201904010473) and Foundation of Guangzhou Science and Information Technology of Guangzhou Key Project (201803040009), Guangzhou Regenerative Medicine and Health Guangdong Laboratory Frontier Research Program (2018GZR110105003), Clinical Innovation Research Program of Guangzhou Regenerative Medicine and Health Guangdong Laboratory (2018GZR0201002), Research Program of the Hefei Institute of Stem Cell and Regenerative Medicine (2019YF001), and Science and Technology Program of Guangzhou (202002020083).

## Conflict of Interest

The authors declare that the research was conducted in the absence of any commercial or financial relationships that could be construed as a potential conflict of interest.

## Abbreviations

CAR, chimeric antigen receptor; PD1, programmed death-1; TIM3, T cell Ig mucin-3; LAG3, lymphocyte activation gene-3; CTLA4, cytotoxic T lymphocyte-associated antigen 4; TME, tumor microenvironment; TAAs, tumor-associated antigens; MDSC: myeloid-derived suppressor cell.
