# Supplementary material for: Chimeric CTLA4-CD28-CD3z T Cells Potentiate Antitumor Activity Against CD80/CD86–Positive B Cell Malignancies
Source: Front Immunol. 2021 Apr 2;12:642528. doi: 10.3389/fimmu.2021.642528 (PMC8050336; doi:10.3389/fimmu.2021.642528)
Supplement: Supplementary file 1 [file DataSheet_1.docx]

**Supplementary figure 1**


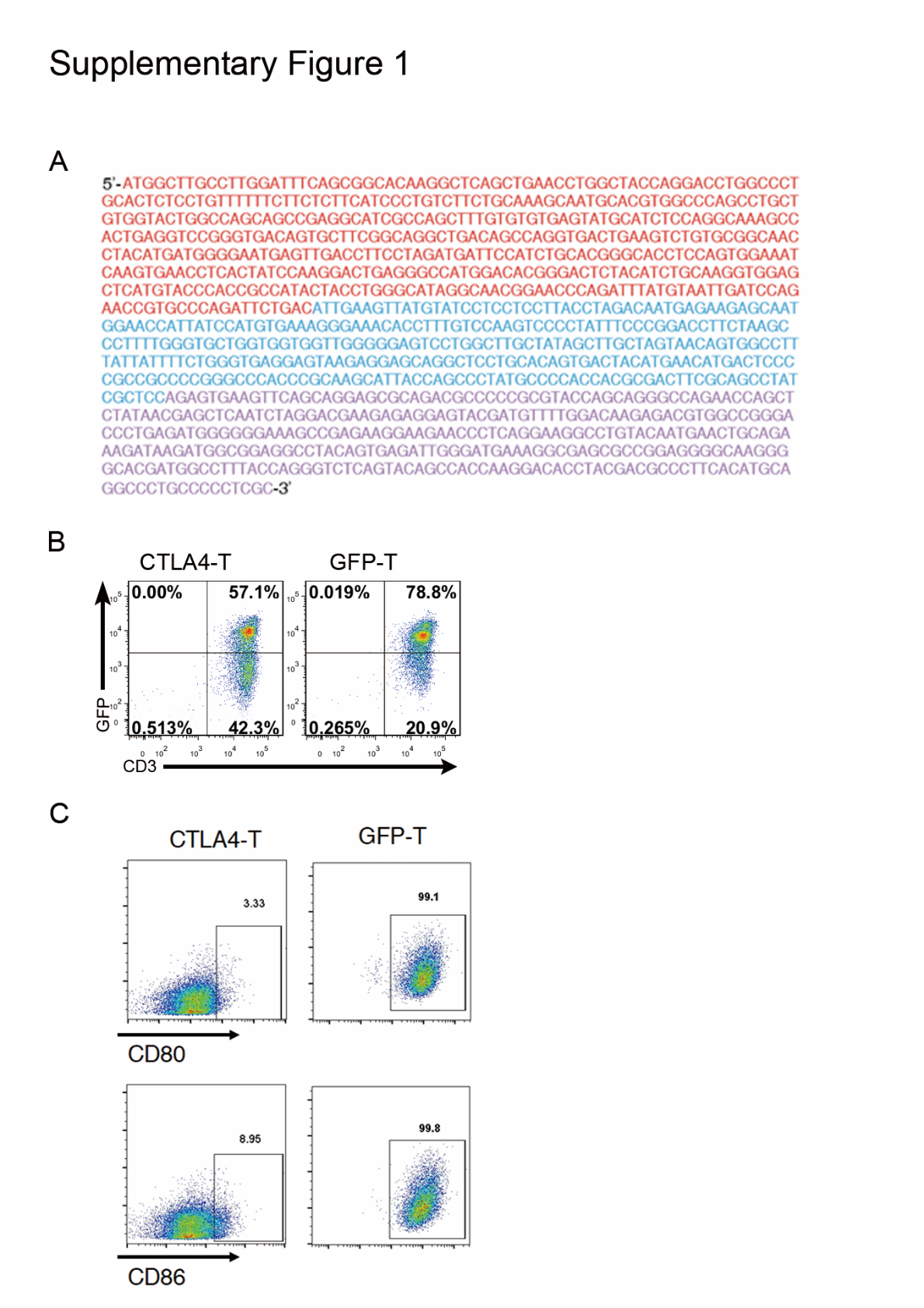


**Supplementary Figure.1 Phenotypic analysis of genetically modified T cells.**

**(A)** The base nucleotide sequence of the human CTLA4 chimeras, which contains the extracellular and transmembrane domains of human CTLA4 (red) and the intracellular domains of human CD28 (blue) and CD3z (purple). **(B)** Representative flow cytometric analysis of GFP expression in T cells transduced with either chimeric CTLA4 or GFP (control). **(C)** Representative flow cytometric analysis of CD80/CD86 expression in Raji tumor cells after coculturing with CAR T cells.

**Supplementary figure 2**


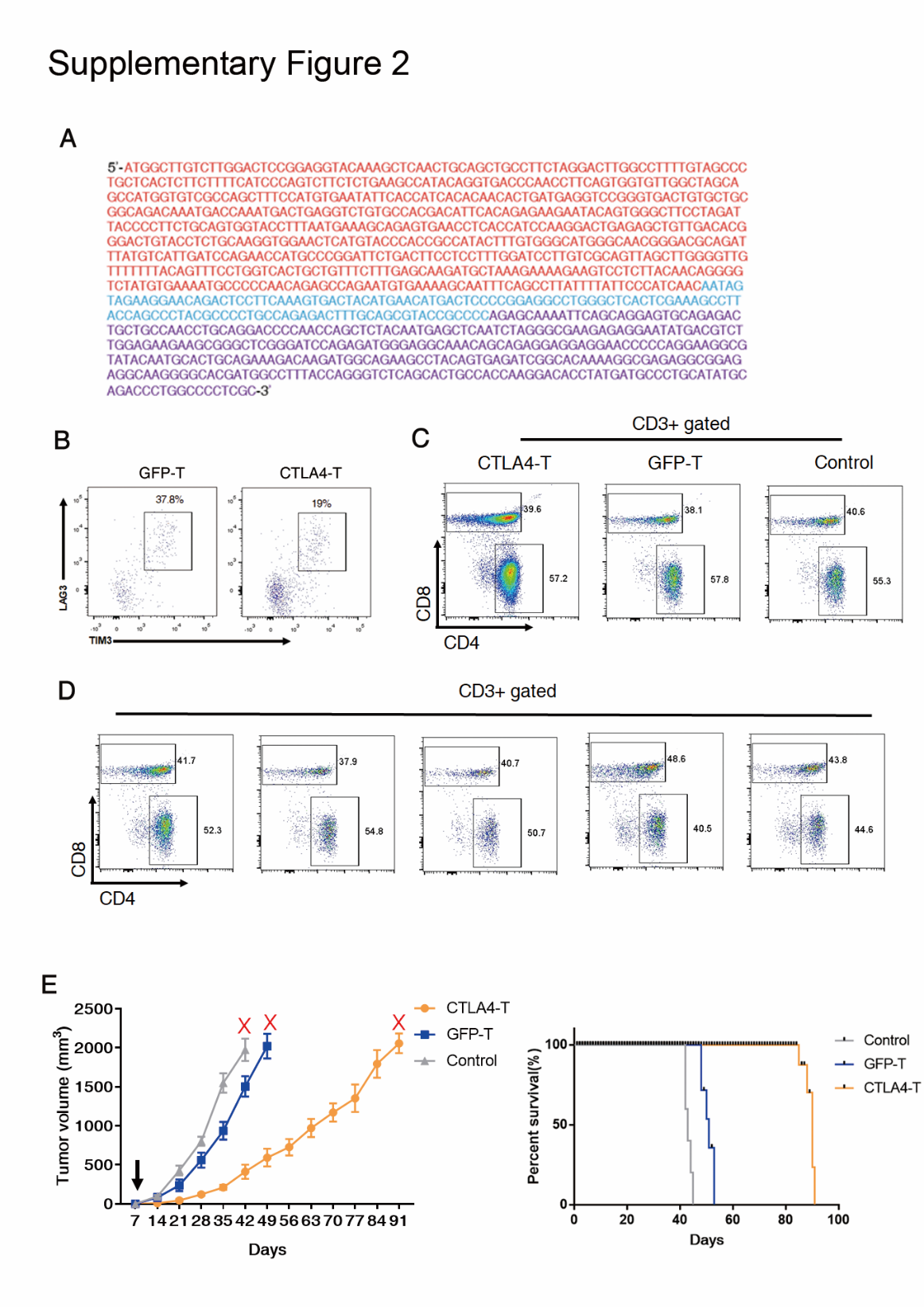


**Supplementary Figure 2. Murine CTLA4-chimeric T cells show toxicity against tumor.**

**(A)** The base nucleotide sequence of the murine CTLA4 chimeras, which contains the extracellular and transmembrane domains of murine CTLA4 (red) and the intracellular domains of mouse CD28 (blue) and CD3z (purple). **(B)** Representative flow cytometric analysis of TIM3 and LAG3 expression in CAR T cells. (C) Representative flow cytometric analysis of CD4 and CD8 expression in CAR T cells before T cell transplantation. (D) Representative flow cytometric analysis of CD4 and CD8 expression in CAR T cells in CTLA4-T group. (E) The B16F10 tumor growth in the mice were measured and calculated every seven days, mice were euthanized once tumor volume exceeded 2000mm^3^, and survival curve of B16F10 implanted mice.
